# Comment on “Three-Principal-Substrate SERS Profiling Enables Reliable Screening of Serum Biomarkers: A General Approach”

**DOI:** 10.1021/acs.analchem.6c01980

**Published:** 2026-06-25

**Authors:** Alois Bonifacio

**Affiliations:** Department of Engineering and Architecture, University of Trieste, Trieste 34127, Italy

## Abstract

In their recent publication, Dong et al. (*Anal. Chem.* **2026**, *98*, 9273; DOI: 10.1021/acs.analchem.5c08093) applied conventional band assignments to interpret serum SERS spectra. However, evidence from our group (Gobbato et al. *Anal. Bioanal. Chem.* **2025**, *417*, 6823; DOI: 10.1007/s00216-025-06192-5) shows that such spectra are dominated by purine metabolites (e.g., uric acid, hypoxanthine), with limited contribution from proteins or lipids. We argue that several assignments in the Dong et al. work may therefore be misleading, overstating biochemical specificity. Rigorous validation of band origins is essential to ensure a reliable interpretation of SERS-based diagnostics.

The recent contribution by Dong et al. in *Analytical Chemistry* ^1^ proposes a multisubstrate SERS strategy to improve the robustness of serum-based diagnostic screening. While the analytical framework is innovative and potentially valuable, we wish to comment specifically on the interpretation of SERS spectral bands, which represents a critical aspect for the biochemical validity of the conclusions.

The band assignments reported in the paper appear to follow common conventions in the SERS biofluid literature, attributing multiple features across the 600–1700 cm^–1^ region to a wide range of biomolecular classes (e.g., proteins, lipids, nucleic acids). However, a growing body of evidence indicates that such generalized assignments may not accurately reflect the true molecular origin of serum SERS signals.

In particular, a recent study by our group^2^ provides a systematic and experimentally validated reassessment of serum SERS spectra under conditions closely matching those used in the *Analytical Chemistry* work (Ag substrates, near-infrared excitation). That study, presenting serum spectra that closely match those reported by Dong et al. (Figure 2, Ag-6%Au, and Figure 3), demonstrates that the observed spectra are dominated by a limited number of low-molecular-weight metabolites, most notably uric acid and hypoxanthine, whose adsorption affinity to silver surfaces governs spectral composition. Through controlled experiments, including standard addition and spectral matching, we show that many bands commonly attributed to proteins (e.g., amide I/III) or lipids do not originate from those biomolecules, under typical SERS conditions.

In this context, the assignments presented in the *Analytical Chemistry* paper raise concerns. Several bands interpreted as arising from complex biomolecular classes are more consistently explained, based on current evidence, by purine metabolites or other strongly adsorbing small molecules. As a result, the biochemical interpretation of the spectral differences—and, by extension, the implied biomarker specificity—may be overstated.

Importantly, this issue does not diminish the potential utility of the proposed multisubstrate approach for classification purposes. However, it suggests that the observed diagnostic performance may rely on variations in a restricted set of metabolites rather than on broad, molecule-specific fingerprints of serum composition. Without experimentally validated band assignments, there is a risk of conflating statistical discrimination with a mechanistic understanding.

We therefore encourage the authors and the community to incorporate rigorous validation of spectral assignments into future SERS studies of biofluids. Such efforts are essential to ensure that advances in analytical performance are accompanied by accurate biochemical interpretation, ultimately strengthening the reliability and translational relevance of SERS-based diagnostics.
